# Beyond Total Error: An Observational Comparative Study of Sigma Metrics in Long-Term Arterial Blood Gas Analyzer Performance

**DOI:** 10.7759/cureus.77236

**Published:** 2025-01-10

**Authors:** Hussien Hamid, Mousa Al-Wafi, Mohamed H Ahmida, Abdulla M Elmansoury, Mohamed Najah

**Affiliations:** 1 Clinical Laboratory Sciences, Libyan International Medical University, Benghazi, LBY; 2 Basic Medical Sciences, Libyan International Medical University, Benghazi, LBY

**Keywords:** accuracy, analytical performance, arterial blood gas analyzers, sigma metrics, total error

## Abstract

Introduction: Accurate respiratory and metabolic status evaluation requires valid and reliable arterial blood gas (ABG) analyzers. This study aimed to evaluate the analytical performance of three (ABG) analyzers using total error (TE) and sigma metric (SM) to establish a robust framework for clinical device performance monitoring.

Methods: The performance of three ABG analyzers (A, B, and C) was evaluated using third-party quality control materials from Randox® across three levels for pH, PCO₂, and PO₂. Each level was analyzed 20 times per analyzer over five days. TE and SM were calculated using standard formulas, with total allowable error (TEa) derived from the 2024 Clinical Laboratory Improvement Amendment (CLIA) guidelines. Mean values were compared to manufacturer-provided target ranges, and performance was assessed against accuracy specifications and Sigma metrics.

Results: All analyzers achieved acceptable mean values within target ranges. Accuracy (TE < TEa) was met for PO₂ and PCO₂ across all analyzers but failed for pH in analyzer A (levels 1, 2), analyzer B (all levels), and analyzer C (levels 1, 3). Sigma metrics showed suboptimal performance for pH (1.6-2.06), PCO₂ ranged from Unaccepted (1.61) to Good (4.34), and PO₂ indicated Good to World-Class performance. No significant differences were observed among analyzers for SM or TE (p > 0.05), but accuracy strongly correlated with performance consistency (Cramér's V = 0.918, p < 0.001).

Conclusion: This study underscores the limitations of relying solely on TE and highlights the importance of integrating SM to ensure reliable and clinically relevant ABG analyzer evaluations.

## Introduction

Blood gas analysis is an essential diagnostic tool that provides insight into a patient's physiological status by evaluating key arterial blood parameters [1]. It plays a critical role in identifying and managing severe conditions such as diabetic ketoacidosis, exacerbations of chronic obstructive pulmonary disease (COPD), and respiratory failure [2].

The clinical laboratory serves as a cornerstone of the healthcare system, as physicians' decisions regarding patient care predominantly rely on the accuracy and reliability of laboratory reports [3]. Clinical laboratory reports play a crucial role in healthcare, influencing 70-75% of medical diagnoses. Among pre-analytical errors, "improper volume" (41.5%) and "undue clotting" (32.8%) were most frequent, with inpatients experiencing these issues more often than outpatients [4]. So, reliable ABG results are crucial for precise clinical decisions, as they guide critical interventions like oxygen therapy adjustments and ventilation management, directly impacting patient outcomes [5].

Observed total error (TEobs) represents the sum of random error (imprecision) and systematic error (bias) for a specific instrument or method [6]. Total error provides a limited perspective on the assessment process, as it depends on a linear model and benefits from being supplemented with the measure of uncertainty (MU) approach. The linear model of TEa faces criticism for its simplistic approach, combining random and systematic errors directly, which overlooks the complexities inherent in real-world laboratory operations [7].

Total allowable error (TEa) specifies the maximum error permissible for laboratory assays, providing a benchmark for method validation, patient or instrument comparison, and quality control design. These performance thresholds ensure assay reliability and consistency within acceptable limits during evaluation and ongoing monitoring [8].

The Six Sigma model was first introduced to clinical laboratories by Nevalainen et al. to evaluate the performance of an analytical process [9]. As an important parameter for assessing the analytical performance of laboratories, the sigma metric (SM) has a significant advantage in quantitative evaluation [10]. Once the analytical performance of the laboratory achieves Six Sigma, there are only 3.4 errors per one million test results and the detection capability of the laboratory has reached the "world-class" level [11].

The Six Sigma model mainly comprises three variables: (TEa), bias, and coefficient of variation (CV). Bias reflects the trueness of analytes, and CV reflects the imprecision of analytes, representing the analytical performance of the laboratory analytical system. However, TEa is closely related to the quality goal selected by the laboratory and is not directly related to the analytical performance of the analytical system itself [10].

Despite recent criticisms regarding the application of Six Sigma in clinical laboratories [12,13], Recent studies highlight the growing application and benefits of analytical sigma metrics. A 2024 survey on quality control (QC) practices in the Netherlands identified three distinct levels of QC implementation: the first relies on the analytical characteristics of measurement procedures, the second incorporates Six Sigma principles by selecting control rules based on sigma-metrics, and the third adopts a risk-based approach that extends Six Sigma methodologies [14].

Building on these advancements, this work aimed to evaluate and compare the analytical performance of three (ABG) analyzers, emphasizing their capability to sustain reliability over time. This assessment integrates (TE) and (SM) methodologies to identify the most robust framework for monitoring and ensuring consistent device performance.

## Materials and methods

Study objective and hypothesis

The study aimed to evaluate the analytical performance of three arterial blood gas (ABG) analyzers in determining the potential of hydrogen (pH), partial pressure of carbon dioxide (PCO₂), and partial pressure of oxygen (PO₂) using Total Error (TE) and Sigma Metric (SM) as quality indicators. We hypothesized that integrating TE with SM would provide a robust framework for consistent performance monitoring, allowing for more comprehensive assessments of analytical reliability.

Study design and justification

This observational comparative study was conducted in Benghazi, Libya, in June 2024. To ensure impartiality, analyzers were anonymized as A, B, and C, with their selection based on established performance in clinical laboratory settings. Third-party quality control (QC) materials were employed due to their ability to mimic clinical sample matrices, enabling reliable assessment of bias, imprecision, and accuracy. This design was chosen to facilitate a realistic evaluation of analyzer performance under typical operating conditions, minimizing intervention biases and capturing the inherent variability present in clinical laboratory environments. While commutability was not explicitly tested, it was assumed based on the materials' design and their widespread use in quality assurance programs, as supported by Badrick et al. [15].

The study focused on two key endpoints. The primary endpoints included assessing compliance with accuracy standards (TE < TEa) and classifying Sigma Metrics for each analyte into categories such as Unacceptable, Good, and World-Class. Secondary endpoints involved comparing the performance of the analyzers and examining the relationship between accuracy and performance consistency using Cramér's V.

Research ethics

Ethical approval for this study was obtained from the Ethical Committee of Libyan International Medical University under the reference number MHS-15-O-00283. Subsequently, the university coordinated with the hospitals housing the devices under evaluation, securing their approval to conduct this research. This study did not involve patient samples, records, or data; all information was derived exclusively from quality control procedures and analyzer performance metrics.

Specimens and experimental procedure

Third-party control materials at three concentration levels (Levels 1, 2, and 3) were obtained from Randox® Laboratories Ltd. (Northern Ireland) for quality control assessment. The catalog and lot numbers for each level were: BG5001-269BG (Level 1), BG5002-284BG (Level 2), and BG5003-286BG (Level 3).

All control samples were prepared, stored, and handled in strict compliance with Randox® guidelines, including storage at 2-8°C and protection from light to ensure sample integrity. Each analyzer was calibrated by highly trained and experienced personnel, following the manufacturer's guidelines. After calibration, each level of control material was tested 20 times on each analyzer over five days, generating a total of 60 readings per analyzer.

Calculation of total errors and sigma metrics

The calculation of the following parameters was performed according to Westgard's basic QC practice [16] using Excel software. For each QC level, the mean values were computed and compared to the reference values to assess systemic deviation (bias). The standard deviation (SD) was calculated and normalized by dividing it by the mean, followed by multiplying it by 100 to derive the coefficient of variation percentage (CV%). To quantify imprecision, both the SD and CV% were subsequently multiplied by 1.65, yielding the absolute imprecision and the percentage imprecision, respectively. TE for each level was calculated as both an absolute value and a percentage by summing the absolute bias with imprecision and the bias percentage with the imprecision percentage.

For the calculation of the SM, two distinct formulas were utilized depending on the parameter's TEa value. For pH at all levels and PCO₂ at Levels 1 and 2, the formula SM = (TEa−Bias)/ SD was applied. For all other parameters and levels, the formula SM = (TEa%−Bias%)/CV% was used. The TEa values employed in this study were derived from the 2024 updates to the CLIA guidelines [17].

Subsequently, the observed mean values for each quality control level and analyzer were compared to the corresponding target ranges provided by the manufacturer to assess compliance with acceptance criteria. TE, both absolute and percentage, for each level and the analyzer was then compared to the respective total error allowance TEa to determine whether the analyzers met the accuracy specifications. Finally, the accuracy for each level and analyzer was evaluated concerning the corresponding Sigma metric to assess its overall analytical performance. The interpretation of Sigma metrics is illustrated in Table 1 [18]. IBM SPSS, version 25.0, was utilized for further statistical analysis. The Kruskal-Wallis test assessed differences in performance among the analyzers, while the Mann-Whitney U test compared performance levels classified as "Passed" and "Not Passed." Receiver operating characteristic (ROC) analysis evaluated the predictive accuracy of Sigma metrics in distinguishing acceptable from unacceptable performance levels. 

**Table 1:** Interpretation of sigma metrics Reproduced from Hamid H et al. [18]. Open access: Permission granted.

Sigma value	Indication
σ value ≥ 6	World-class performance
σ value ≥ 5	Excellent performance
σ value ≥ 4	Good Performance
σ value ≥ 3	Marginal Performance
σ value ≥ 2	Poor Performance
σ value < 2	Unacceptable performance

## Results

The pH accuracy and analytical performance varied significantly across the analyzers. analyzer A achieved acceptable mean pH values within the target ranges across all quality control (QC) levels, as shown in Table 2. Total errors at Levels 1 and 2 slightly exceeded the allowable limits (TEa ±0.04 mmHg). With sigma metrics of 1.6, 1.7, and 1.99, the overall performance was classified as unacceptable (Table 3). Similarly, analyzer B reported mean pH values within target ranges but showed consistent inaccuracies, with total errors surpassing the limits at all levels (0.06 mmHg, 0.05 mmHg, and 0.08 mmHg) (Table 2). This resulted in sigma metrics of 0.73, 1.48, and 0.65 and an overall unacceptable classification (Table 3). Analyzer C displayed mean pH values within target ranges (Table 2); however, total errors at Levels 1 and 3 exceeded the TEa, measuring 0.043 mmHg and 0.06 mmHg, respectively. While Level 2 showed acceptable accuracy with a total error of 0.039 mmHg and a sigma metric of 2.06 (minimally acceptable), Levels 1 and 3 were classified as unacceptable based on sigma metrics of 1.86 and 1.27, respectively (Table 3).

**Table 2:** Comparison of observed mean values and target ranges across ABG analyzers at different QC levels ABG: Arterial blood gas; QC: Quality control

Test Parameter	Q.C Level	Target Value	Target Range	Mean±SD	Observed Range
Analyzer A					
pH	Level 1	7.099	(7.001-7.197)	7.106 ± 0.02	(7.046 - 7.166)
	Level 2	7.414	(7.332-7.496)	7.405± 0.02	(7.345 - 7.465)
	Level 3	7.545	(7.483-7.607)	7.545± 0.02	(7.485 - 7.605)
PCO₂	Level 1	75.01	(60.00-90.01)	76.01± 1.68	(70.97 - 81.05)
	Level 2	39.53	(31.67-47.40)	39.04± 1.49	(34.57 - 43.51)
	Level 3	20.027	(16.05-24.00)	19.861± 1.34	(15.841 - 23.881)
PO₂	Level 1	114.01	(85.51-142.51)	113.4± 2.52	(105.84 - 120.96)
	Level 2	124.51	(105.76-143.26)	122.4± 3.49	(111.93 - 132.87)
	Level 3	144.762	(123.01-166.51)	140.28± 2.35	(133.23 - 147.33)
Analyzer B					
pH	Level 1	7.099	(7.001-7.197)	7.125± 0.019	(74.873 - 74.987)
	Level 2	7.414	(7.332-7.496)	7.442± 0.022	(39.214 - 39.346)
	Level 3	7.545	(7.483-7.607)	7.526± 0.031	(20.727 - 20.913)
PCO₂	Level 1	75.01	(60.00-90.01)	74.93± 1.44	(70.61 - 79.25)
	Level 2	39.53	(31.67-47.40)	39.28± 1.37	(35.17 - 43.39)
	Level 3	20.027	(16.05-24.00)	20.82± 1.05	(17.67 - 23.97)
PO₂	Level 1	114.01	(85.51-142.51)	109.65± 2.37	(102.54 - 116.76)
	Level 2	124.51	(105.76-143.26)	125.65± 4.15	(113.2 - 138.1)
	Level 3	144.762	(123.01-166.51)	144.45± 3.25	(134.7 - 154.2)
Analyzer C					
pH	Level 1	7.099	(7.001-7.197)	7.099± 0.021	(7.036 - 7.162)
	Level 2	7.414	(7.332-7.496)	7.41± 0.018	(7.356 - 7.464)
	Level 3	7.545	(7.483-7.607)	7.55± 0.028	(7.466 - 7.634)
PCO₂	Level 1	75.01	(60.00-90.01)	78.57± 1.59	(73.8 - 83.34)
	Level 2	39.53	(31.67-47.40)	39.5± 1.43	(35.21 - 43.79)
	Level 3	20.027	(16.05-24.00)	20.08± 1.14	(16.66 - 23.5)
PO₂	Level 1	114.01	(85.51-142.51)	112.4± 2.8	(104 - 120.8)
	Level 2	124.51	(105.76-143.26)	125.4± 4.12	(113.04 - 137.76)
	Level 3	144.762	(123.01-166.51)	143.1± 2.99	(134.13 - 152.07)

**Table 3:** Comparison of accuracy and sigma metric performance across ABG analyzers and QC levels ABG: Arterial blood gas; QC: Quality control

Test Parameter	Q.C Level	Bias	Bias%	TE	TE%	TEa	TEa%	Accuracy	Sigma Metric	Performance
Analyzer A										
pH	Level 1	0.01	0.1	0.05	0.67	± 0.04	-	Not Passed	1.6	Unaccepted
	Level 2	0.01	0.14	0.05	0.66	± 0.04	-	Not Passed	1.7	Unaccepted
	Level 3	0	0	0.04	0.52	± 0.04	-	Passed	1.99	Unaccepted
PCO₂	Level 1	1	1.33	4.28	5.65	-	8	Passed	3.02	Marginal
	Level 2	0.49	1.24	3.41	8.71	± 5.0	-	Passed	3.03	Marginal
	Level 3	0.17	0.83	2.80	14.04	± 5.0	-	Passed	3.6	Marginal
PO₂	Level 1	0.61	0.53	5.55	4.89	-	15	Passed	6.51	World-Class
	Level 2	2.11	1.69	8.94	7.28	-	15	Passed	4.67	Good
	Level 3	4.48	3.1	9.08	6.38	-	15	Passed	7.12	World-Class
Analyzer B										
pH	Level 1	0.03	0.37	0.06	0.90	± 0.04	-	Not Passed	0.73	Unaccepted
	Level 2	0.01	0.11	0.05	0.68	± 0.04	-	Not Passed	1.48	Unaccepted
	Level 3	0.02	0.07	0.08	0.87	± 0.04	-	Not Passed	0.65	Unaccepted
PCO₂	Level 1	0.08	0.11	2.91	3.88	-	8	Passed	4.1	Good
	Level 2	0.25	0.64	2.94	7.48	± 5.0	-	Passed	3.46	Marginal
	Level 3	0.79	3.96	2.84	13.81	± 5.0	-	Passed	4.02	Good
PO₂	Level 1	4.36	3.82	9.0	8.06	-	15	Passed	5.17	Excellent
	Level 2	1.14	0.92	9.27	7.38	-	15	Passed	4.27	Good
	Level 3	0.31	0.22	6.69	4.63	-	15	Passed	6.57	World-Class
Analyzer C										
pH	Level 1	0	0	0.042	0.59	± 0.04	-	Not Passed	1.86	Unaccepted
	Level 2	0	0.05	0.04	0.52	± 0.04	-	Passed	2.06	Poor
	Level 3	0.01	0.07	0.06	0.78	± 0.04	-	Not Passed	1.27	Unaccepted
PCO₂	Level 1	3.56	4.75	6.67	8.71	-	8	Not Passed	1.61	Unaccepted
	Level 2	0.03	0.08	2.84	7.19	± 5.0	-	Passed	3.47	Marginal
	Level 3	0.05	0.24	2.29	11.39	± 5.0	-	Passed	4.34	Good
PO₂	Level 1	1.61	1.41	7.09	6.29	-	15	Passed	5.46	Excellent
	Level 2	0.89	0.72	8.97	7.16	-	15	Passed	4.35	Good
	Level 3	1.66	1.15	7.52	5.24	-	15	Passed	6.63	World-Class

For PCO₂, analyzer A demonstrated acceptable mean values across all QC levels, with the total error percentage (TE%) at Level 1 within limits but accompanied by a sigma metric of 3.02, indicating marginal performance (Table 2). Levels 2 and 3 showed improved accuracy yet remained marginal, with sigma metrics of 3.03 and 3.6, respectively. Analyzer B displayed strong accuracy with TE% values well below the allowable TEa% threshold of 8% at all levels. However, sigma metrics varied: good at Level 1 (4.1), marginal at Level 2 (3.46), and good at Level 3 (4.02), reflecting performance variability (Table 3). Analyzer C reported acceptable mean values within target ranges across all QC levels (Table 2). However, at Level 1, the total error exceeded the allowable limit (8.98%), leading to an unacceptable sigma metric of 1.61. Levels 2 and 3 performed better, with TE values below 5.0 mmHg and sigma metrics of 3.47 (marginal) and 4.34 (good), respectively, showing improved performance at higher QC levels (Table 3).

The PO₂ performance was consistently satisfactory across analyzers, with mean values remaining within target ranges at all QC levels (Table 2). Analyzer A maintained accuracy with total errors within the allowable TEa of 15%, achieving world-class sigma metrics at Levels 1 (6.51) and 3 (7.12) and good performance at Level 2 (4.67). Similarly, analyzer B demonstrated reliable accuracy with total errors below the 15% threshold across all levels. Sigma metrics reflected excellent performance at Level 1 (5.17), good performance at Level 2 (4.27), and world-class classification at Level 3 (6.57). Analyzer C also reported acceptable mean values within target ranges, with total errors below 15% at all levels. The sigma metrics highlighted excellent performance at Level 1 (5.46), good performance at Level 2 (4.35), and world-class performance at Level 3 (6.63), showcasing consistent accuracy and precision (Table 3).

Correlation between accuracy and long-term performance levels in ABG analyzers

The accuracy pass rates evaluated by TE for analyzers A, B, and C were 77.78%, 66.67%, and 66.67%, respectively. All analyzers demonstrated marginal performance based on average sigma metrics of 3.69, 3.38, and 3.45, respectively.

The Kruskal-Wallis test revealed no significant differences in sigma metric and total error values across three analyzers, with p-values of 0.897 and 0.917, respectively. A statistically significant and strong correlation was identified between accuracy and performance consistency over time (Cramér's V = 0.918, p < 0.001). 

**Figure 1:**
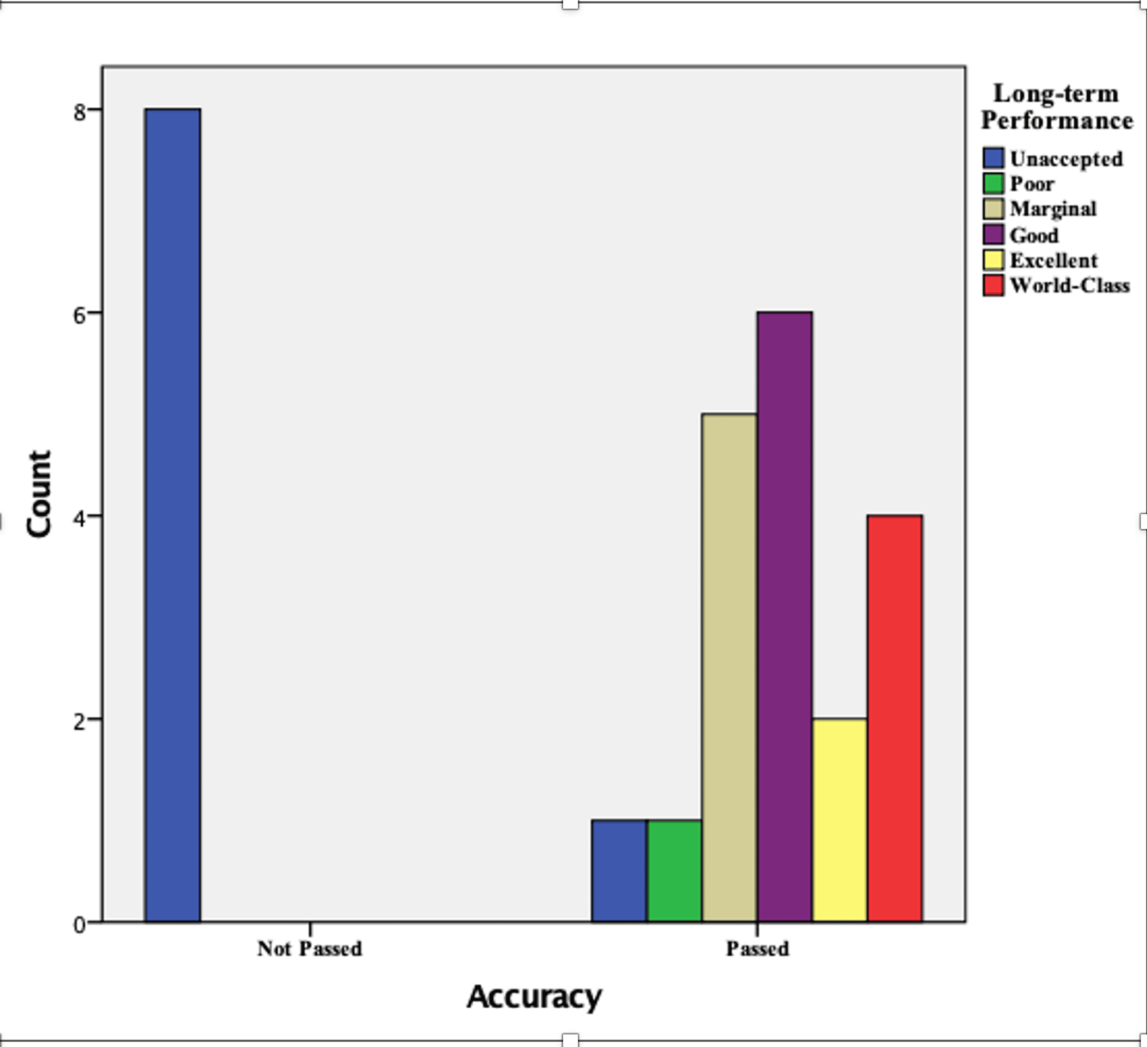
Distribution of performance categories based on accuracy compliance

Based on the Pearson chi-square analysis (χ² = 22.737, p < 0.001), all quality control levels categorized as "Not Passed" for accuracy were exclusively associated with unacceptable performance (Figure 1). Conversely, quality control levels that met accuracy criteria exhibited a diverse distribution across performance categories: one was classified as poor, five as marginal, six as good, two as excellent, and four achieved world-class performance. Additionally, the Mann-Whitney U test revealed a significant difference in sigma metric levels between quality control (QC) levels categorized as 'Passed' and 'Not Passed,' with a p-value of 0.001.

**Figure 2:**
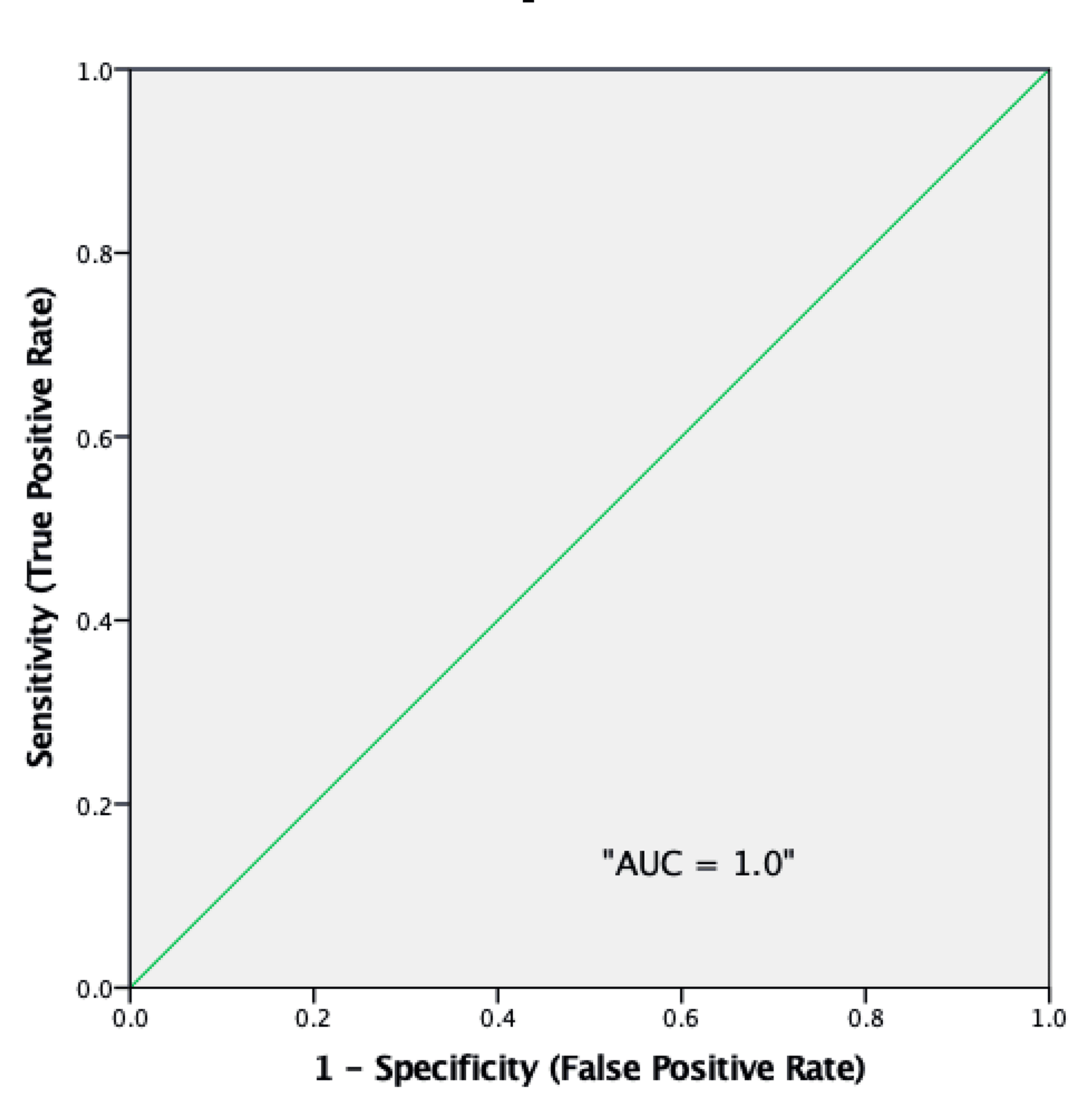
Receiver operating characteristic curve for sigma metrics in accuracy prediction AUC: Area under the curve

The receiver operating characteristic (ROC) analysis yielded an area under the curve (AUC) of 1.0, indicating that the SM is a perfect predictor of accuracy, with no overlap or ambiguity between accurate and inaccurate cases (Figure 2).

## Discussion

This study aimed to evaluate and compare the analytical performance of three arterial blood gas (ABG) analyzers, focusing on their capability to sustain reliability over time. The performance of each analyzer was assessed using two key performance indicators: TE and SM, to identify the most robust framework for monitoring consistent device performance in clinical settings.

All levels across all analyzers achieved target mean values, Table 2, with TE accuracy pass rates of 77.78%, 66.67%, and 66.67% for analyzers A, B, and C, respectively, Table 3. While most of the QC levels met the criteria of (TE<TEa), SM results demonstrate significant variability Table 3. This raises critical concerns regarding the adequacy of TE alone as a reliable indicator for assessing the quality of laboratory results. This concern aligns with the observations of Oosterhuis (2017), who highlighted several limitations of the TE approach. He noted that estimating TE represents only one aspect of the assessment process and emphasized that TE relies on a linear model and should be complemented by the measure of uncertainty (MU) approach [7]. 

Despite the great reputation of Six Sigma, particularly in the health sector, its application in the field of clinical laboratories has recently received wide criticism. The applicability of the Sigma Metrics (SM) equation in clinical laboratories has been questioned by Badrick and Theodorsson (2024), who have argued that it has failed to introduce any truly innovative or practical solutions to laboratory medicine. Instead, it has merely sparked debates over existing methodologies [12]. This criticism is further supported by Coskun and Oosterhuis (2024), who emphasized that incorrect calculation of the SM often leads to an underestimation of process performance, raising concerns about its reliability and effectiveness [13]. 

In response, Hassan Bayat and colleagues (2024) robustly advocate for the efficacy of SM, countering criticisms by emphasizing its pivotal role in reducing variability, optimizing costs, and enhancing patient safety through streamlined laboratory processes. By integrating systematic and random errors into a unified performance metric, SM serves as a precise and practical tool for evaluating and enhancing laboratory quality. Furthermore, it supports the development of comprehensive Quality Control (QC) strategies by enabling laboratories to select optimal QC rules and frequencies tailored to risk levels and performance requirements [14].

Six Sigma can help determine the optimal Westgard rules by evaluating process performance against a reference method, thereby identifying areas in laboratory processes that need improvement [19]. In a study by Joseph Litten (2017) evaluating the effectiveness of a Sigma metric-based QC program, a clear inverse relationship was observed between Sigma performance levels and QC failure rates. Methods at 6 Sigma achieved the lowest failure rate of 0.7% across 30,044 tests, while those at 5 Sigma had a higher failure rate of 2.1% over 10,875 tests. Methods at 3 and 4 Sigma exhibited the highest failure rate, with 14.7% recorded across 5,572 tests [20]. These findings highlight the pivotal role of higher Sigma metrics in improving analytical reliability and reducing QC failures.

In this study, While the TE < TEa measure offers a broad measure of accuracy, Sigma metrics exposed significant performance variability among analyzers, even for parameters classified as "accurate" under TEa criteria. This observation validates the role of Sigma metrics as a necessary assessment, capable of bridging the gaps in traditional accuracy-centric evaluations by incorporating precision and reliability dimensions.

Regarding the differences in sigma metrics and total error values between the three analyzers, the Kruskal-Wallis test showed no significance (p-values of 0.897 and 0.917, respectively), suggesting similar overall performance, which is consistent with findings from Roels et al. [21]. However, this lack of significant variance in statistical terms does not negate the nuanced differences in performance observed over time. For instance, in this study, the QC levels that failed accuracy criteria (TE > TEa) showed unacceptable" sigma performance, while those meeting accuracies varied from poor to world-class, Figure 1. 

Furthermore, the Kruskal-Wallis test showed a significant difference in Sigma metric levels between QC levels categorized as "Passed" and "Not Passed" (p-value = 0.001), the Mann-Whitney U test highlights the critical role of Sigma metrics in assessing performance over time. By differentiating between acceptable and unacceptable levels of accuracy, Sigma metrics effectively highlight their role in capturing both accuracy and precision-essential factors for ensuring long-term reliability in clinical diagnostics and improving patient outcomes. This perspective aligns with insights from Kaplan et al. [22], who emphasize that precise measurement tools significantly influence patient-centered outcomes, including treatment success and recovery pathways.

The AUC of 1.0 obtained from ROC analysis demonstrates that the Sigma Metric (SM) is an exceptional predictor of accuracy, exhibiting no overlap or ambiguity between accurate and inaccurate cases, Figure 2. This perfect classification ability highlights Sigma metrics' potential to serve as a cornerstone for performance evaluation, addressing both present and predictive analytical quality measures [18].

In light of the foregoing, the observed strong and statistically significant correlation between SM and TE values (Cramér's V = 0.918, p < 0.001) represents a notable and critical finding. This highlights the critical role of continuous monitoring of device performance and how long-term reliability is closely linked to consistent accuracy. By reinforcing this link, the results suggest a symbiotic relationship where improvements in one domain (accuracy) bolster the other (precision). This finding aligns with earlier arguments by Hassan Bayat and colleagues [14], emphasizing the capability of SM to detect systematic and random errors in a single performance measure.

Based on our results and inspired by the renowned adage, "All models are wrong, but some models are useful" [23], as well as the 2021 recommendations from the Accredited Medical Laboratories Network (LABAC) in France [15], the SM has demonstrated exceptional value. This tool has empowered laboratory supervisors to effectively evaluate long-term performance quality and optimize the use of reagents and internal quality control (I.Q.C) materials, particularly in high-volume laboratories. These outcomes align with the observations of Heerden et al. [19].

Limitations 

The limited testing duration and the number of QC measurements constrain the evaluation of long-term analyzer stability and performance trends. To enhance the clinical relevance of the findings, future studies should incorporate real patient samples, as this study exclusively relied on third-party QC materials. Additionally, the use of Total Error Allowable (TEa) values from the 2024 CLIA guidelines may reduce the generalizability of the results to non-CLIA settings where different regulatory or clinical standards might apply.

## Conclusions

In summary, while TEa provides an essential measure of overall accuracy, the findings from this study highlight the importance of incorporating Sigma metrics for a more comprehensive performance evaluation. Sigma metrics not only supplement accuracy-focused criteria but also enable the early identification of inconsistencies, offering actionable insights to maintain high analytical standards. This dual approach strengthens the assessment of ABG analyzers and underscores the importance of adopting a multidimensional framework that evaluates both accuracy and precision, ensuring the reliability of clinical devices in routine use. Future research should aim to integrate Sigma metrics into routine quality control across clinical settings, assess long-term benefits, and evaluate cost implications, with the potential for improved device reliability and reduced error-related costs justifying initial investments.
